# Effectiveness and Feasibility of Self-Monitoring for Weight Management in Individuals With Mental Disorders Using Digital Intervention: Protocol for a Stepped-Wedge Cluster Randomized Trial (“SWIM” Study)

**DOI:** 10.2196/78420

**Published:** 2026-04-27

**Authors:** Jiamiao Wu, Jinjie Xu, Jingyi Bi, Xuequan Zhu, Le Xiao

**Affiliations:** 1 Beijing Key Laboratory of Intelligent Drug Research and Development for Mental Disorders, National Clinical Research Center for Mental Disorders, National Center for Mental Disorders, Beijing Anding Hospital, Capital Medical University, Ankang lane No.5, Beijing, 100088, China

**Keywords:** mHealth, digital health, weight management, serious mental illness, stepped-wedge trial, obesity, metabolic syndrome, self-monitoring, lifestyle intervention, mobile health

## Abstract

**Background:**

Individuals with schizophrenia or bipolar disorder face a significantly elevated risk of obesity, primarily due to weight gain associated with psychiatric medications and lifestyle factors. While digital self-monitoring tools offer scalable solutions, their application remains underexplored in psychiatric populations. To address these gaps, this type 1 hybrid effectiveness-implementation study investigates the preliminary effectiveness and implementation feasibility of a mobile health-assisted weight management intervention for patients with severe mental illness.

**Objective:**

This study aims to evaluate the preliminary effectiveness and implementation feasibility of a mobile health–assisted weight management intervention for patients with severe mental illness transitioning from inpatient care to community-based recovery.

**Methods:**

This single-center, open-cohort stepped-wedge cluster randomized trial with a 2-month step duration will recruit 204 patients from 6 clinical units. Clusters are randomized into 2 waves, with staggered transitions to a digital intervention, including smart scales, health apps, and biweekly educational modules, over a 6-month observation period. The design evaluates the intervention across the transition from inpatient care to community-based recovery. The primary outcome is the proportion of participants achieving ≥5% weight loss at month 6. Implementation feasibility is assessed through device technical success and intervention adherence (defined as ≥50% completion of weekly weigh-ins and daily dietary logs).

**Results:**

Participant data collection began in May 2023 and was completed by June 2025 with a total of 204 participants. The publication of key findings and results is anticipated in late 2026.

**Conclusions:**

This protocol describes a pragmatic, technology-supported intervention designed to address metabolic side effects in a tertiary psychiatric setting. By bridging the gap between acute hospitalization and community recovery, this hybrid stepped-wedge cluster randomized trial provides a crucial framework for integrating digital metabolic monitoring into routine clinical workflows for vulnerable populations.

## Introduction

### Background

Patients with severe mental illness (SMI) face a 2‐3-fold higher prevalence of obesity and metabolic syndrome than the general population, contributing to 15‐20 years reduced life expectancy [1-3]. These comorbidities lead to elevated cardiovascular risk, impaired treatment adherence, and diminished social functioning [2,4-6]. This metabolic burden is driven by a complex interplay of factors, primarily the metabolic side effects of antipsychotic medications, compounded by sedentary lifestyles and poor dietary habits [7-9].

Although China is undergoing rapid mental health reform, metabolic monitoring in patients with SMI remains insufficient. Available data suggest that the prevalence of comorbid obesity among patients with schizophrenia in China is approximately 16.4% [10]; however, systematic metabolic screening is rarely implemented in routine psychiatric care. Globally, treatment guidelines have long recommended regular physical health monitoring for patients with SMI; only 38% of them receive guideline-concordant metabolic screening [11,12]. The National Institute for Health and Care Excellence has issued updated clinical guidelines advocating mandatory annual BMI surveillance as a preventive measure for individuals with chronic health conditions [13]. This recommendation aligns with emerging epidemiological evidence demonstrating that systematic BMI monitoring reduces obesity-related complications by 22% (95% CI 15%‐28%) in populations with long-term multimorbidity [14].

Digital health technologies have emerged as a particularly promising avenue for delivering these interventions. Recent meta-analyses indicate that comprehensive digital programs incorporating nutritional tracking, exercise prescription, and peer support can achieve a clinically meaningful mean weight reduction of 3.2 (95% CI 2.1‐4.3) kg over 3 months in SMI populations, with notably higher retention rates than traditional in-person interventions [15]. Mobile health (mHealth) tools, in particular, have demonstrated superior adherence and weight loss outcomes compared to paper-based methods [16], and comparable outcomes compared to in-person programs [17,18]. However, significant limitations and gaps in the current evidence base remain, which are particularly relevant when considering the generalizability of findings to Chinese psychiatric settings. Many existing studies are characterized by small sample sizes that preclude examination of clinical heterogeneity and the identification of patient-level predictors of success [19,20]. Furthermore, there is an overreliance on step-counting wearables that neglect other critical metrics like body composition and dietary intake [19]. Crucially, the follow-up durations in most trials are insufficient to assess the long-term sustainability of weight loss and behavioral changes [18,20].

The evidence specific to Chinese SMI populations is even scarcer. While digital health is rapidly expanding in China, most mHealth studies for chronic disease management have excluded or underrepresented individuals with SMI, overlooking the unique cognitive, motivational, and socioeconomic barriers this group faces [21,22]. The bidirectional mental health impacts of digital weight management also remain unclear and potentially complex. While some studies report ancillary benefits such as reduced depressive symptoms following mHealth interventions (β=.31, *P*=.02) [23], others warn of potential harms, including exacerbated body image concerns or anxiety related to constant self-monitoring [20]. These mixed findings underscore the need for careful implementation and evaluation.

To address these challenges, we designed an mHealth-assisted intervention specifically targeting antipsychotic-induced obesity in a Chinese SMI population and evaluated it using a stepped-wedge cluster randomized trial (SW-CRT) to balance rigor with pragmatic implementation.

**Figure 1.**
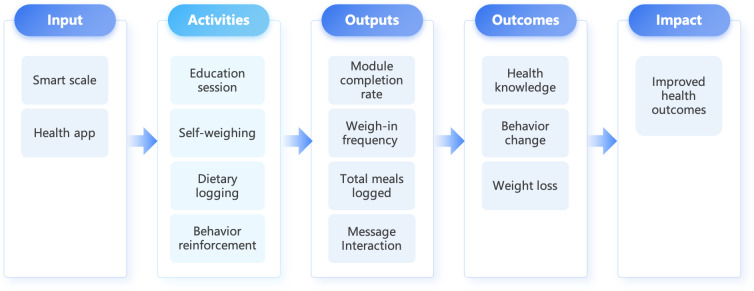
Theoretical framework and logic model of the intervention.

The SWIM intervention is grounded in social cognitive theory (SCT) and self-regulation principles, which suggest that behavior change arises from dynamic interactions between personal, behavioral, and environmental factors [21,22,24]. Specifically, the intervention targets core SCT constructs, self-monitoring, self-efficacy, and observational learning, using digital tools like food diaries and biometric tracking to provide necessary feedback for behavioral adjustment [25,26]. Clinical adaptations address the specific cognitive and behavioral barriers faced by individuals with schizophrenia and bipolar disorder [27]. The theoretical pathway from digital inputs to clinical impact is illustrated in Figure 1. The framework delineates the pathway from technical inputs (smart scales and mobile apps) to clinical impact. Specifically, it highlights how core activities, such as educational sessions and biometric tracking, generate measurable outputs, including weigh-in frequency, dietary logging completion, and app engagement, which serve as the behavioral foundation for improving health knowledge and fostering self-efficacy. These intermediate changes are hypothesized to drive the primary outcome of weight loss and the long-term impact on overall health. To examine the mechanism of action, this trial will measure self-monitoring frequency and self-efficacy as key process variables. We hypothesize that improvements in these constructs will mediate the intervention’s effect on weight loss.

The model illustrates the progression from digital inputs and activities to measurable implementation outputs (engagement) and clinical outcomes, mediated by SCT constructs.

This trial uses a type 1 hybrid implementation-effectiveness design to concurrently evaluate clinical outcomes and implementation feasibility within a stepped-wedge framework.

### Primary Objective

The primary objective is to evaluate the effectiveness of the digital intervention on body weight reduction. The primary outcome is the proportion of participants achieving ≥5% weight loss at the end of the intervention phase compared to the control phase. This threshold, along with the ≥7% threshold, is selected based on established evidence linking these levels of weight loss to significant cardiovascular and metabolic health benefits in the SMI population [28-31]. Based on recent trials of tailored digital interventions in similar populations, which reported ≥5% success rates ranging from 15% to 36.6% [32], we anticipate that the intervention will achieve a success rate of at least 40%, significantly higher than the expected 20% success rate in the control phase.

### Secondary Objectives

Secondary objectives focus on capturing granular metabolic improvements and assessing implementation feasibility. To fully evaluate metabolic change beyond binary thresholds, we will analyze absolute changes in body weight (kg) and BMI as continuous outcomes. Additional clinical assessments include changes in fasting blood glucose, lipid profiles, and self-reported psychosocial functioning. Feasibility is evaluated through device technical success (smart scale pairing rates), participant adherence (frequency of weekly weigh-ins and dietary logs), and digital usability scores. We hypothesize that the intervention will demonstrate good feasibility, defined as ≥50% adherence to weekly weigh-ins, and that higher adherence will correlate with greater improvements in clinical and metabolic outcomes.

### Exploratory Objectives

As a hypothesis-generating component, we will explore individual-level predictors of clinical success and engagement, including digital literacy, baseline psychopathology, and medication adherence. These exploratory analyses will be adjusted for multiple comparisons to minimize the risk of type I error.

## Methods

### Study Design

This study uses a pragmatic, open-cohort SW-CRT with a 2-month step duration. The trial spans a total observation window of 6 months across 6 clinical units at a tertiary hospital in Beijing. The SW-CRT design ensures that all participants eventually receive the intervention, fulfilling ethical requirements for this high-risk population, while the staggered rollout allows the implementation team to maintain high fidelity by providing intensive technical training for 1 wave at a time.

The trial addresses the dynamic turnover of psychiatric hospitalizations (average stay 4‐6 wk) by defining each cluster as a clinical unit rather than a fixed physical location. The research team comprises trained research assistants, certified nutritionists, and sports physicians. Participants are typically enrolled during their stable recovery phase in the ward; however, the intervention is designed to be portable. Following discharge, the intervention continues remotely, with the same clinical team providing longitudinal oversight via digital clinician dashboards. This approach enables the evaluation of the intervention across the transition from inpatient care to community-based recovery.

The 6 units were randomized into 2 waves (3 units per wave) using a matched-pair constrained randomization approach. To ensure baseline balance, units were paired based on clinical setting and demographics. Allocation was managed by an independent biostatistician via REDCap (Research Electronic Data Capture; Vanderbilt University) and remained concealed from the recruitment staff and participants.

The study timeline is structured into three distinct phases:

1. Baseline phase (month 1): all clusters receive usual care to establish stable baseline metrics.
2. Transition phase (2 weeks): a staggered 2-week period for device distribution and technical training, occurring at month 1 for wave 1 and month 3 for wave 2.
3. Intervention phase: active digital monitoring lasting 5 months for wave 1 (months 2-6) and 3 months for wave 2 (months 4-6).

**Figure 2.**
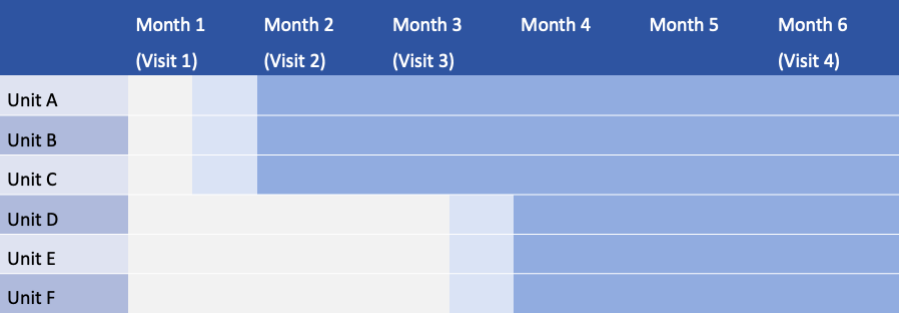
Stepped-wedge design matrix across six clinical clusters over 6 months. The figure illustrates the staggered transition from usual care (gray) to the digital intervention phase (blue) following a 2-week transition period (light blue).

This open-cohort approach accounts for the dynamic turnover of psychiatric hospitalizations, allowing for individual enrollment while maintaining randomization at the cluster level (Figure 2).

### Patient Recruitment

Due to the dynamic turnover of psychiatric hospitalizations, an open-cohort approach was adopted, allowing for continuous recruitment throughout the study period. Recruitment follows a structured clinician-referral model across the 6 clinical units to ensure both clinical appropriateness and implementation fidelity. The process is organized into the following stages: Treating psychiatrists identify potential participants during routine clinical encounters in the outpatient and inpatient departments. Patients are considered for referral if they meet three prerequisites: (1) a BMI ≥24 kg/m², (2) a demonstrated interest in weight management, and (3) a confirmed stable clinical phase, defined as a minimum of 4 weeks on a stable medication regimen.

Upon referral, an independent project physician conducts a formal in-person screening session. This involves a review of the patient’s electronic health records (EHR) to confirm *ICD-10* (*International Statistical Classification of Diseases, Tenth Revision*) diagnoses and physical measurements of height, weight, and blood pressure. To ensure the validity of historical data, the exclusion criterion regarding significant prior weight loss is ascertained through a multi-informant approach, cross-referencing patient interviews with collateral reporting from family caregivers and EHR data. Detailed inclusion and exclusion criteria are summarized in Textbox 1.

Textbox 1. **Inclusion and exclusion criteria.**

**Inclusion criteria**

- Age 18-60 years.
- *ICD-10 (International Statistical Classification of Diseases, Tenth Revision*) diagnosed schizophrenia or bipolar disorder.
- Overweight (BMI ≥24 kg/m² is defined as overweight or obese according to Chinese national standards).
- Current use of ≥1 antipsychotic or mood stabilizer (eg, lithium and valproate).
- Owning a smartphone and capable of reading.
- Expecting to remain in the study throughout the entire study period.

**Exclusion criteria**

- Inability to provide informed consent in line with ethical requirements.
- ≥7% weight loss in the past 6 months.
- “Secondary obesity” (such as hyperthyroid).
- The weight >150 kg.
- Pregnancy or planning pregnancy.
- Cardiovascular contraindications.
- Current participation in other trials.

### Informed Consent and Capacity Assessment

Due to the dynamic turnover of psychiatric hospitalizations (average stay 4‐6 wk), we use a consecutive, open-cohort recruitment model. Treating psychiatrists identify potential participants during routine clinical encounters. To prevent anticipation bias, crossover dates are disclosed to each unit’s implementation team only 2 weeks prior to their scheduled transition. This separation of roles ensures that the recruitment process remains objective and independent of the unit’s study phase. Given the vulnerability of the SMI population, a structured capacity assessment is conducted prior to enrollment. Participants must demonstrate comprehension of the study’s procedures, data workflows, and the right to withdraw. Written informed consent is obtained from all participants, with primary family caregivers strongly encouraged to witness the process and co-sign the form to provide an additional layer of advocacy. Signed documents are encrypted and stored securely at the research center.

To systematically assess the representativeness of the study sample, a comprehensive screening log is maintained for all identified patients. We record de-identified demographic and clinical data (age, sex, primary diagnosis, and EHR-derived BMI) for all screened candidates, including those excluded due to lack of smartphone access, weight limits (>150 kg), or refusal to participate. At the trial’s conclusion, a comparative analysis between enrolled participants and the broader hospital population will be performed to quantify recruitment bias and characterize the digital divide within the SMI population.

### Intervention Program

The SWIM intervention is a multicomponent digital health program grounded in SCT, designed to enhance self-regulation for weight management. Participants are provided with a Huawei Pro 3 Bluetooth-enabled smart scale and the Huawei Health App (v10.0.2) installed on their personal smartphones. The intervention is coordinated by a multidisciplinary research team, including a trained research assistant for technical setup, a certified nutritionist for dietary content, and a psychiatrist for clinical oversight.

The program follows a hybrid delivery model, initiating in the psychiatric inpatient units of Beijing Anding Hospital and continuing remotely in the participants’ community settings post discharge. During the 2-week transition period, participants receive in-person training on biometric tracking and dietary logging. The intervention lasts for the duration of the assigned wave (5 months for wave 1 and 3 months for wave 2). Participants are instructed to perform weekly weigh-ins and daily dietary logging. To support behavioral change, biweekly educational modules are delivered via WeChat (Tencent) groups. See Multimedia Appendix 1 for the smart scale setup guide and weighing protocol. Furthermore, weekly automated push notifications and personalized feedback messages are sent via messages, with content tailored to each participant’s specific weight trajectory (Table 1).

**Table 1.** Summary of SWIM intervention components.

Components	Delivery method	Frequency
Smart scale weigh-ins	Digital Health App and compatible smart scale	Weekly (at least once per week)
Dietary logging	Digital Health App	Daily (all meals and snacks)
Education session	WeChat groups	Biweekly
Behavior enhancing	Message prompts and tips	Weekly (Automated push notifications and personalized feedback)

### Outcomes and Measures

#### Overview

This study uses a Type 1 hybrid implementation-effectiveness framework to concurrently evaluate clinical outcomes and implementation feasibility. Assessments are conducted at Baseline (month 0) and follow-up intervals at months 1, 2, 3, and 6 (Multimedia Appendix 2). Data collection integrates EHR, laboratory tests, and structured assessments. Baseline assessments take approximately 60 minutes, with 25-minute follow-ups. To ensure long-term sustainability, we use a tiered engagement protocol for nonadherence: automated reminders, followed by counselor outreach (after 2 wk), and finally in-person support for persistent cases. Technical support is provided via video tutorials and a dedicated hotline.

Financial incentives are avoided to favor clinically translatable behavior reinforcement strategies. To address the risk of multiplicity and ensure statistical rigor, we have established a clear hierarchy of endpoints.

To address the risk of multiplicity and ensure statistical rigor, we have established a clear hierarchy of endpoints.

#### Primary Outcome

The primary effectiveness outcome is the proportion of participants achieving ≥5% weight reduction from baseline, assessed at the end of the 6-month study period (month 6). This allows for a final assessment across both waves, regardless of their crossover wave.

#### Secondary Outcomes

To address the limitations of binary variables and capture broader metabolic health improvements, we have established a clear hierarchy of secondary endpoints:

1. Key clinical markers: these include the absolute change in body weight (kg) and BMI, body fat percentage, and metabolic markers (fasting blood glucose and lipid profiles). Weight metrics are automatically recorded via the Huawei Pro 3 smart scale.
2. Implementation feasibility: feasibility is measured by device pairing success rate and intervention adherence. While participants are instructed to perform weekly weigh-ins and daily dietary logging, “Good adherence” for analysis purposes is operationally defined as completing ≥50% of expected weekly weigh-ins (ie, at least 2 weigh-ins per month) and logging dietary intake on ≥50% of days during the intervention period. Acceptability is assessed via the System Usability Scale (SUS) and semistructured interviews with 30 purposely selected participants (Multimedia Appendix 3).
3. Psychosocial and functional outcomes: we use validated Chinese versions of psychometric scales with established reliability (Cronbach α>0.80) [33-42]:

- Clinical symptoms: Patient Health Questionnaire-9 (PHQ-9) for depression, Generalized Anxiety Disorder-7 (GAD-7) for anxiety, Brief Psychiatric Rating Scale Version 4.0 (BPRS-4) for acute psychopathology, and Mood Disorder Questionnaire (MDQ) for mood swings.
- Functioning and quality of life: Sheehan Disability Scale (SDS) for functional impairment and Quality of Life Enjoyment and Satisfaction Questionnaire-Short Form (Q-LES-Q-SF) for life satisfaction.
- Cognitive and physical well-being: Perceived Deficits Questionnaire–Depression, 5-item (PDQ-D5) for subjective cognitive deficits, Chalder Fatigue Scale (CSF-11), Perceived Stress Scale (PSS), and Arizona Sexual Experience Scale (ASEX).
- Weight motivation: a 0‐100 mm Visual Analogue Scale (VAS) quantifies the intent to lose weight.

### Ethical Considerations and Data Security

This study was conducted in strict accordance with the Declaration of Helsinki and received formal ethical approval from the Ethics Committee of Beijing Anding Hospital (Approval No. [2022] Research [202]-202321FS-2). Given the vulnerability of the SMI population, a structured, multistage informed consent process was implemented. Potential participants underwent a capacity assessment by their treating psychiatrist to ensure they understood the study’s nature and risks. We used the Teach-back method, where participants were asked to explain the study’s core components in their own words to verify comprehension. For participants with partial capacity, written informed consent was obtained from both the participant and their legally authorized representative (primary caregiver).

To mitigate risks associated with the use of a proprietary app (Huawei Health), we have implemented a data-decoupling protocol. Researchers do not have access to participants’ cloud accounts or backend data. Instead, de-identified metrics (weight and BMI only) are submitted via encrypted WeChat Enterprise Edition (TLS 1.3) channels. Redacted data are stored on password-protected hospital servers using Advanced Encryption Standard (AES)-256 encryption with 2-factor authentication. Access logs are maintained for auditing, and all study-specific digital data will be permanently deleted 6 months post-completion, adhering to the minimum necessary period principle.

To address the risk of technological failure (eg, server downtime or software bugs), a manual backup procedure is in place. Participants are instructed to read weight values directly from the smart scale’s LED display and record them in a provided paper log or via encrypted messaging. Paper-based food diaries will serve as a contingency tool for dietary logging. This dual-track system ensures continuity of data collection and minimizes reliance on a single digital interface.

### Adverse Events

Trial safety is overseen by an independent steering committee. Serious adverse events, including life-threatening incidents, psychiatric hospitalizations, or serious violent incidents, are reported within 24 hours. Recognizing the potential for digital-specific harms in the SMI population, we have transitioned from a reactive to a preventive monitoring framework. A dedicated data safety officer reviews unblinded adherence data monthly. “Red flag” patterns, such as obsessive weighing (>3 times/day) or a sudden cessation of logging after high engagement, will trigger an immediate clinical welfare check. We explicitly monitor for increased anxiety surrounding weigh-ins, worsening body image concerns, and digital fatigue. The emotional experience of app usage will also be explored during semi-structured exit interviews with a subset of participants. Should psychosocial distress be identified, a predefined supportive protocol is activated, including counseling to reframe the intervention as a supportive tool and practical adjustments to monitoring frequency. The digital intervention will be discontinued for any participant demonstrating (1) a clinically significant increase in psychiatric symptoms (eg,> 20% increase in BPRS-4 scores), (2) the emergence of technology-related delusions, or (3) new-onset disordered eating behaviors. All adverse events will be assessed for their relationship to the trial in accordance with HRA requirements and reported in the final study results.

### Intervention Checkpoints

The intervention checkpoints are presented in Table 2.

**Table 2.** Intervention checkpoints.

Components	Checkpoints
Participants	Setup: successfully install the study app and pair the Bluetooth-enabled smart scale. Core usage: demonstrate competency in weight logging and food diary recording. Perform weekly self-weighing and dietary tracking as instructed. Follow-up compliance: complete lab tests and surveys at designated visits upon receiving notifications.
Study app	Data visualization: upon data entry (weight and diet), the app generates weight trajectory charts and dietary gap rainbow. Data security: automatically encrypt and transfer response data to HIPAA^a^-compliant servers.
Research team	Routine motivation: send scheduled external reminders (via phone messages) to prompt weekly weighing and dietary recording. Retention: monitor the clinician dashboard for inactivity. Initiate counselor calls or clinic visit invitations if participants miss >2 scheduled weigh-ins. Visit: manually track and notify participants monthly to complete required questionnaires and lab tests.

a HIPAA: Health Insurance Portability and Accountability Act.

### Sample Size Estimation

The sample size was powered based on the mean reduction in body weight (kg) to provide greater statistical sensitivity than binary outcomes. Drawing from meta-analyses of digital interventions for SMI [8] and the recent CoachToFit trial [32], a Cohen *d* of 0.40 was identified as a clinically realistic target effect size. Using PASS 2021 (stepped-wedge cluster-randomized design module) with 6 clusters and monthly repeated measurements, a total sample of 204 participants (34 per unit) provides 82% power (α=.05, 2-tailed) assuming a conservative intracluster correlation coefficient (ICC) of 0.10. This value is consistent with empirical benchmarks for metabolic outcomes in psychiatric institutional settings [43-45]. Furthermore, this sample size is sufficiently robust to detect the primary categorical outcome of ≥5% weight loss, assuming a 20% success rate in the control phase and a 40% success rate in the intervention phase (odds ratio≈2.6). Accounting for a 20% attrition rate (aligned with recent SMI trial observations), the final evaluable sample (n=163) maintains ≥75% power even under varying cluster performance, ensuring robust detection of the treatment effect under pragmatic constraints.

### Statistical Plans

#### Overview

All analyses will follow the intention-to-treat principle. We prioritize effect size estimation over formal hypothesis testing. Implementation outcomes, including recruitment, retention, and adherence rates, will be reported with 95% CIs. For clinical intervention effects, we will report point estimates with 80% CIs to balance precision with exploratory use, as wider intervals better reflect the preliminary nature of these estimates and minimize the risk of overinterpreting early trends. To account for the different intervention durations between waves, intervention exposure duration (time since crossover) will be included as a time-varying covariate in the mixed-effects models. This approach allows for the estimation of the intervention effect while adjusting for the varying lengths of exposure across clusters.

#### Clinical Effectiveness Analysis

To account for the nested cluster structure and secular time trends inherent in the stepped-wedge design, we use 2 complementary modeling approaches. For the primary binary outcome (≥5% weight loss), a generalized estimating equations model with a logit link function will be used to compare proportions across study phases. For continuous secondary outcomes (eg, mean percentage weight change and BMI reduction), we will use a Linear Mixed-Effects Model. The model is specified as follows:


Yijk=β0+β1⋅Interventionjk+Σγk⋅Timek+Σδm⋅Covariatesijk+αj+ζij+ϵijk

Where:

- Yijk represents the continuous outcome for individual i in cluster j at time k.
  β1estimates the treatment effect (Intervention vs Control); γk represents categorical secular time trends to isolate the intervention’s impact from time-related fluctuations; and δm denotes fixed-effect adjustments for the prespecified covariates. αj is the random intercept for the cluster (clinical unit) to account for ICC; ζij is the random intercept for the individual to account for repeated measurements over time; and ϵijk represents the residual error.

#### Covariate Adjustment and Confounder Control

Following the framework established by Chinman [32], the models will control for critical pharmacological and clinical confounders. To account for the different intervention durations between waves, intervention exposure duration will be included as a time-varying covariate. Antipsychotic treatments will be categorized into high-risk (eg, clozapine and olanzapine) and low-to-moderate risk groups, also incorporated as time-varying covariates to account for any regimen changes during the 6-month period. To isolate the intervention’s effect from “pseudo-weight loss” caused by medication nonadherence, medication possession ratios will be calculated; participants with a medication possession ratio <80% will be analyzed as a separate subgroup in sensitivity analyses. Additionally, stratified analyses based on baseline weight status (overweight vs obese) will be conducted to explore potential moderating effects.

Missing data will be addressed via multiple imputation under missing at random assumptions. Further sensitivity analyses, including pattern mixture models and tipping point analysis, will be performed to evaluate the impact of potential missing not at random mechanisms. To mitigate the risk of type I error inflation across multiple secondary and subgroup analyses, the false discovery rate correction will be applied. All analyses will be conducted using SAS 9.4, with clinical and feasibility implications prioritized over nominal statistical significance.

### Dissemination

Study results will be released to participants and referring clinicians upon request. Results will be published in scientific journals and conferences targeting mental health professionals and researchers within computer science. The final trial results will be reported in accordance with the CONSORT (Consolidated Standards of Reporting Trials) extension for SW-CRTs.

## Results

The study has progressed as follows: funding was secured in March 2022, the Ethics Approval was guaranteed in August 2022, and participant data collection began in May 2023 and was completed by June 2025, with a total of 204 participants. The follow-up period was finished in January 2026. Initial data analysis is in progress, and the publication of key findings and results is anticipated by late June 2026. The final trial results will be reported in accordance with the CONSORT extension for SW-CRTs.

## Discussion

### Anticipated Findings

Patients with SMI often face significant metabolic side effects and weight gain, which can lead to anxiety, low self-esteem, social isolation, and decreased adherence to treatment, which underscores the importance of implementing effective weight management strategies in this vulnerable population [10,29,30]. The protocol outlines an innovative hybrid stepped-wedge trial for managing antipsychotic-induced obesity using digital tools with multidisciplinary support. This study explores the largely untested use of smartphone-based weight management and smart scale technology in individuals with SMI [18,31,32]. The methodologically rigorous design ensures ethical equity by providing all clusters access to the intervention while enabling robust control of temporal and cluster-level confounders through stratified randomization and time-trend adjustment [33].

Beyond its methodological strengths, the study advances a person-centered care model that fosters self-awareness, autonomy, and active engagement among individuals with SMI [28]. For too long, individuals with SMI have experienced diminished personal agency and inconsistent care [34,35]. By integrating smart devices into routine psychiatric services, this intervention aims to promote continuity of care and support patient engagement, thus empowering individuals to detect early weight changes and take proactive steps toward improving physical health.

While this study uses the Huawei Health ecosystem due to its high market penetration and device synergy in China, the intervention components, self-weighing, dietary logging, and behavioral nudges, are designed based on SCT and are platform-independent. This approach allows for future reproducibility using alternative digital health platforms.

By linking personal digital tools with hospital and community support systems, the intervention promotes collaborative dialog among patients, caregivers, and health care professionals. This may help reduce stigma, improve care continuity, and foster digital literacy—key elements for developing equitable, scalable mental health solutions in resource-limited settings.

Several limitations warrant critical examination regarding the study’s external validity. First, the smartphone ownership requirement introduces a significant selection bias. By excluding individuals without mobile devices, we likely omitted the most socioeconomically marginalized patients and those with the most severe cognitive impairments or functional decline, who are often at the highest risk for metabolic syndrome. Consequently, our findings may overestimate the effectiveness of digital interventions in the broader SMI population where digital literacy and device access remain major barriers.

Second, a potential limitation is the transition from a cluster-based environment to individual remote monitoring post discharge. However, this reflects the real-world scenario, and our use of mixed-effects modeling robustly handles the resulting open-cohort structure and varying exposure durations. Last, the single-center recruitment at a premier tertiary psychiatric hospital in Beijing may constrain generalizability. Participants at such a specialized center may have higher educational levels, better familial support, and access to more comprehensive baseline care than those in rural or community-based settings in China. Therefore, the successful implementation observed here might not be immediately replicable in resource-limited primary care environments. Future multicenter trials involving diverse geographic and socioeconomic regions are essential to validate these findings across the full spectrum of psychiatric care settings. Nonetheless, our findings provide a critical baseline for these future comparative studies.

While we acknowledge the selection bias toward digital-literate patients and the single-center nature of this trial, this study serves as a preliminary proof-of-concept. It explores how national policy goals can be operationally adapted for the psychiatric population—a group often overlooked in general health campaigns. Rather than a finalized standard operating procedure, this trial provides initial evidence on the feasibility of digital self-monitoring. These findings are a critical first step toward developing more inclusive, multicenter strategies that could eventually inform the integration of physical health management into the “Basic Public Health Service” for patients with SMI, provided the digital divide identified in our limitations can be bridged.

### Conclusions

This protocol describes a pragmatic, technology-supported intervention designed to address weight gain associated with psychiatric medications through an open-cohort stepped-wedge framework. By using digital tools to maintain monitoring during the transition from inpatient care to community-based recovery, findings from this hybrid SW-CRT are expected to demonstrate the preliminary effectiveness and practical feasibility of self-monitoring in a tertiary psychiatric setting. This study provides a structured approach to integrating digital metabolic tracking into routine clinical workflows, offering a potentially scalable and sustainable strategy to improve physical health outcomes for individuals with SMI.

## Supplementary material

10.2196/78420 Multimedia Appendix 1 The smart scale user guide for patients.

10.2196/78420 Multimedia Appendix 2 Assessment timeline of the trial.

10.2196/78420 Multimedia Appendix 3 User experience survey on the Huawei Health app and smart scale usage.

10.2196/78420 Multimedia Appendix 4 CONSORT (Consolidated Standards of Reporting Trials) flow diagram.

10.2196/78420 Checklist 1 Detailed description of the intervention using the TIDieR checklist.

10.2196/78420 Checklist 2 SPIRIT checklist.

10.2196/78420 Checklist 3 CONSORT 2010 Extension for Stepped-Wedge Cluster Randomized Trials Checklist.

## Abbreviations

ASEX: Arizona Sexual Experience Scale
BPRS-4: Brief Psychiatric Rating Scale, Version 4.0
CFS-11: Chalder Fatigue Scale-11
CONSORT: Consolidated Standards of Reporting Trials
EHR: electronic health record
GAD-7: Generalized Anxiety Disorder-7
ICC: intracluster correlation coefficient
*ICD-10*: *International Statistical Classification of Diseases, Tenth Revision*
MDQ: Mood Disorder Questionnaire
mHealth: mobile health
PANSS: Positive and Negative Syndrome Scale
PDQ-D5: Perceived Deficits Questionnaire – Depression, 5-item
PHQ-9: Patient Health Questionnaire-9
PSS: Perceived Stress Scale
Q-LES-Q-SF: Quality of Life Enjoyment and Satisfaction Questionnaire-Short Form
REDCap: Research Electronic Data Capture
SCT: social cognitive theory
SDS: Sheehan Disability Scale
SMI: severe mental illness
SUS: System Usability Scale
SW-CRT: stepped-wedge cluster randomized trial
VAS: Visual Analogue Scale
