# Supplementary material for: Effectiveness and Feasibility of Self-Monitoring for Weight Management in Individuals With Mental Disorders Using Digital Intervention: Protocol for a Stepped-Wedge Cluster Randomized Trial (“SWIM” Study)
Source: JMIR Res Protoc. 2026 Apr 27;15:e78420. doi: 10.2196/78420 (PMC13120533; doi:10.2196/78420)
Supplement: Multimedia Appendix 1 [file resprot-v15-e78420-s001.docx]

**体脂秤与手机APP测量体重图示**

**测量姿势**

1、将智能体脂秤放置在坚硬平坦的地面，秤脚无异物、未放在砖缝中。


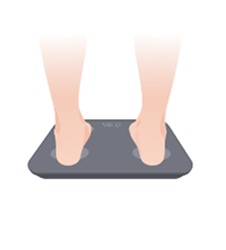

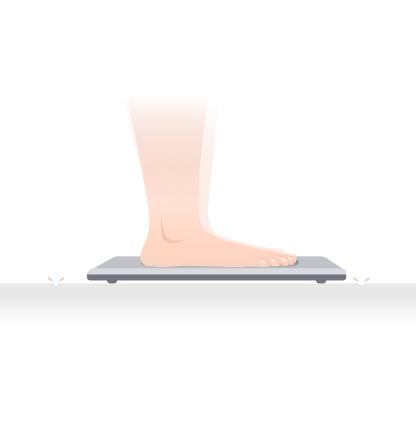


2、保持脚底干燥，秤面洁净，无异物或水渍，穿着贴身薄衣，赤裸双脚站在秤的电极板上进行测量，测量过程中站直并且保持稳定。


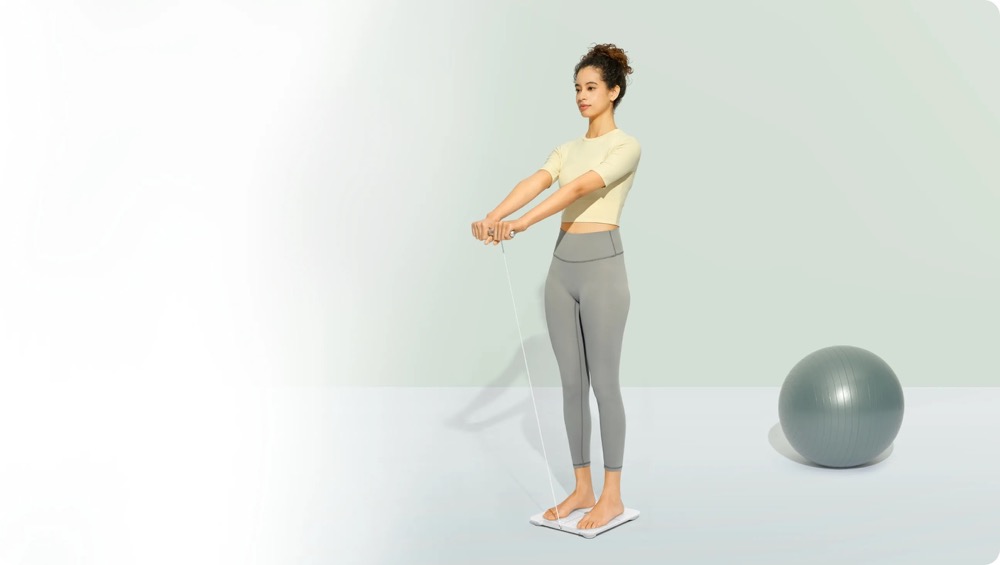


3、测量时，需要拉起拉杆，左手握紧标注L的手柄，右手握紧标注R的手柄，如图所示，胳膊伸直，肘关节伸直，静立20s 左右。

**测量方法**

**1、打开华为运动健康APP主页面**

- 在首页点击**体重管理**卡片，选择测量的用户，点击下方**测量体重**，根据界面提示上秤测量。


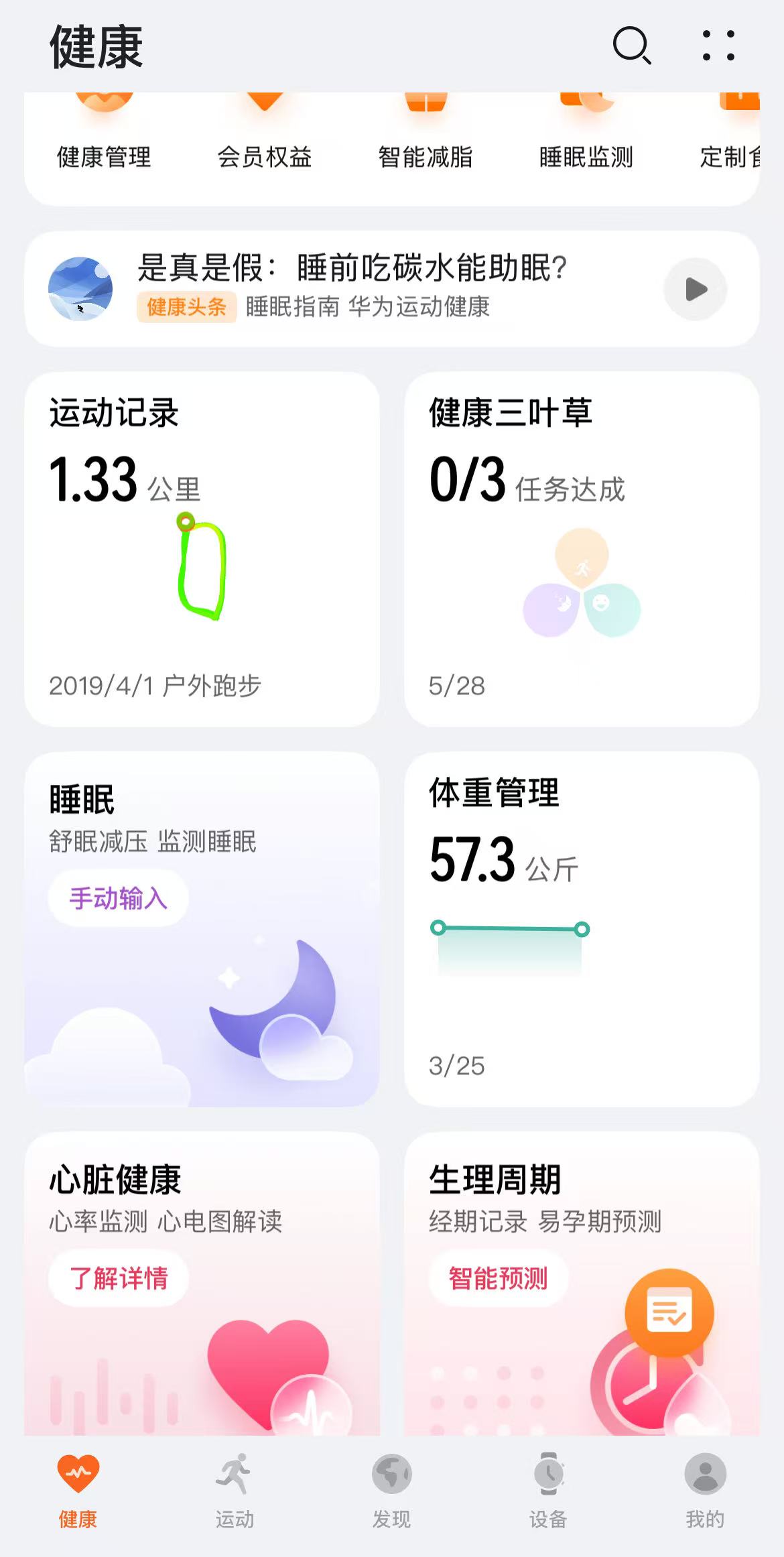

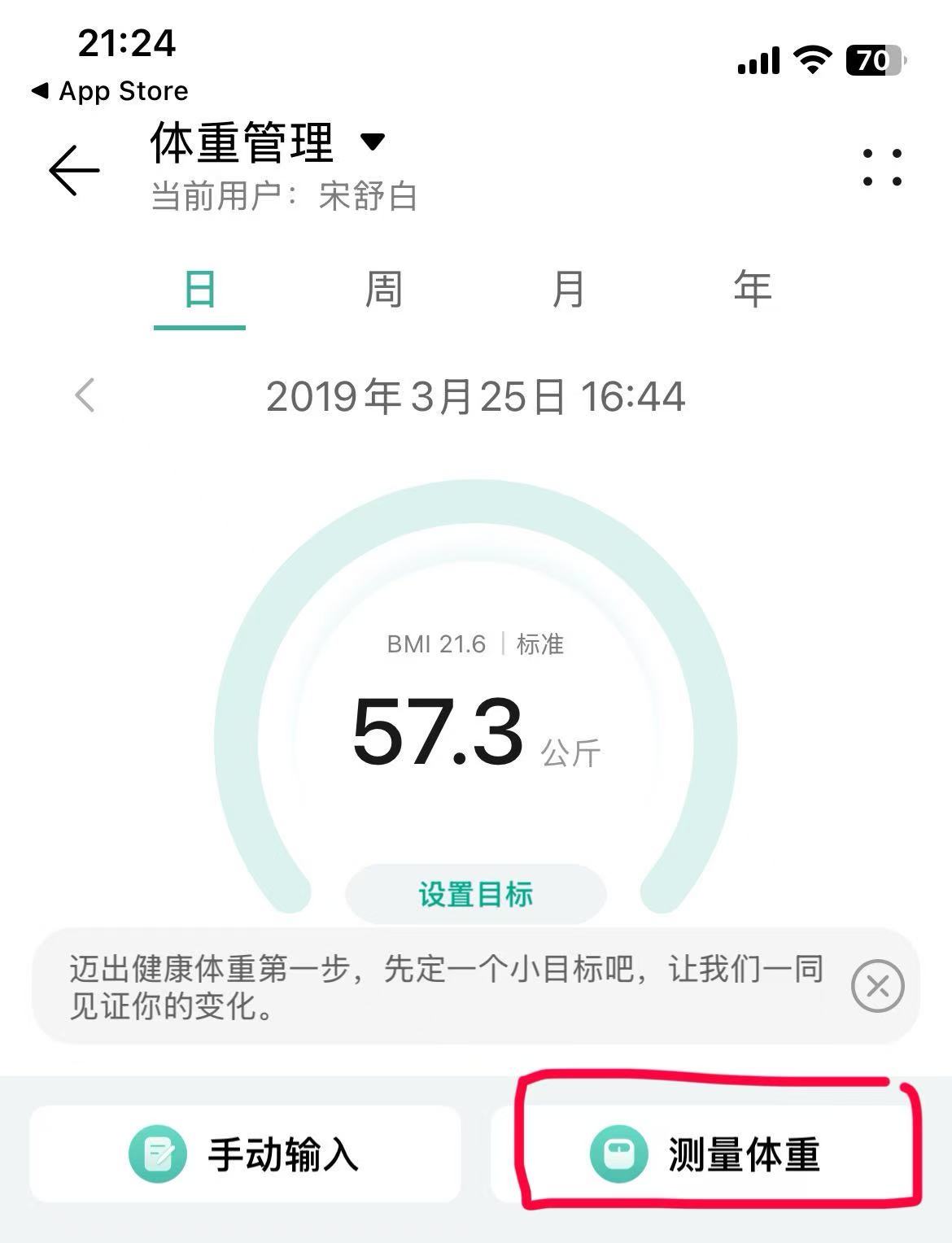

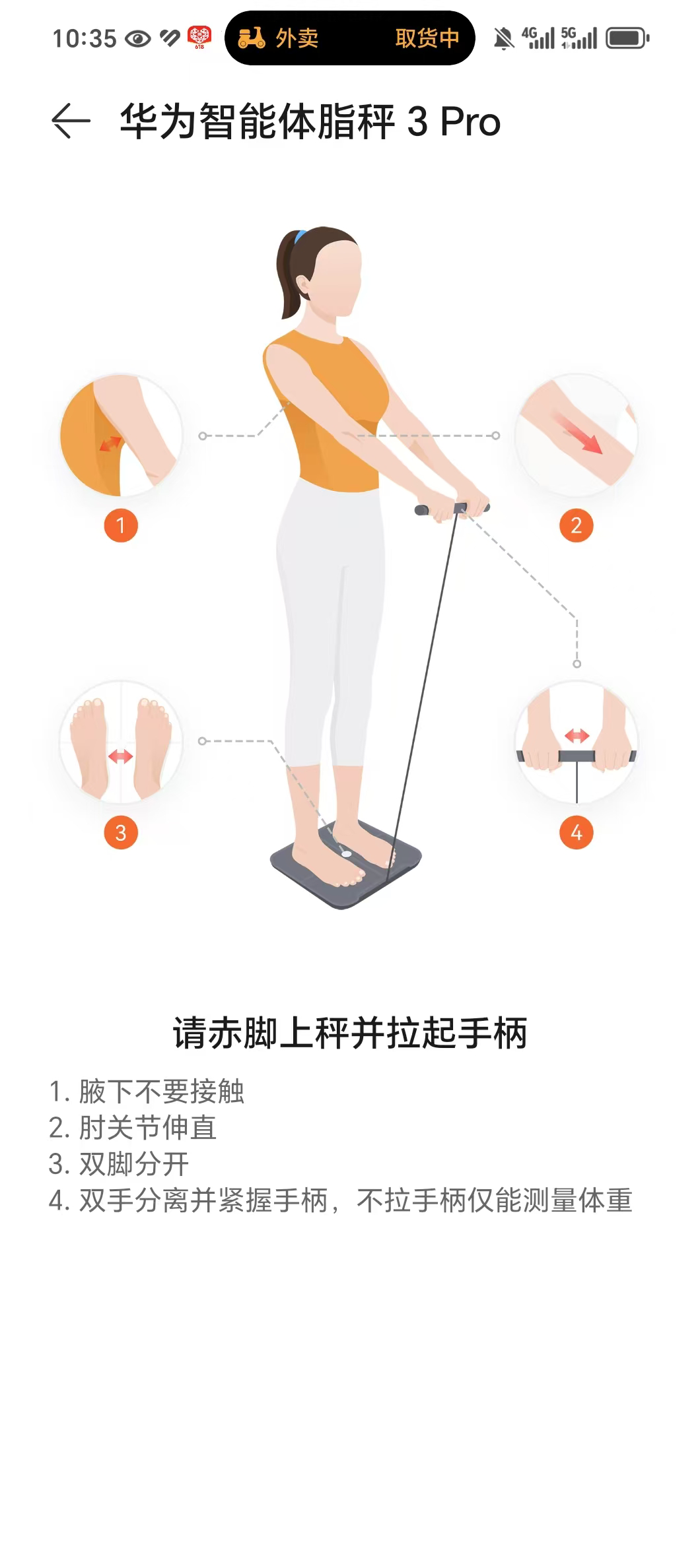


- 在设备页点击已绑定的体脂秤，进入**设备详情页**，点击**开始测量**，根据界面提示上秤测量。

**2、如何提示我测量成功了？**

准备好姿势后只需要站立不动（约10秒），此时称显示屏上会依次出现你的体重、体脂率及心率，若三个数字都闪动了一遍，则代表称重成功，手机上自动会出现以下两种形式的截图。

**
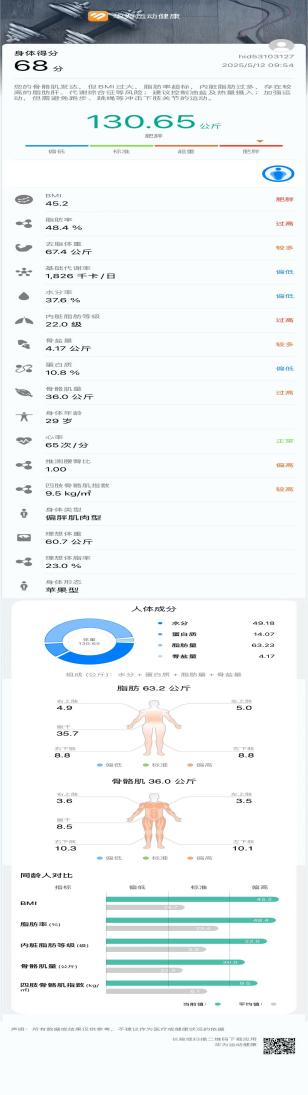

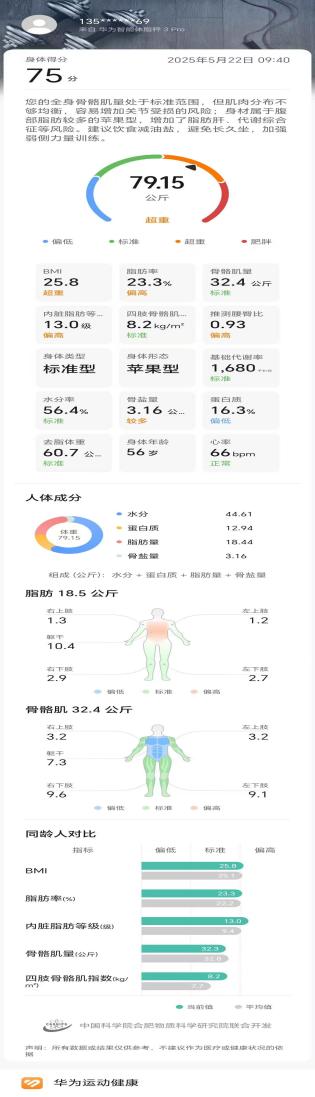
**

图一：IOS系统截图 图二：Android系统截图

**3、称上面闪动的一些图标代表什么？**


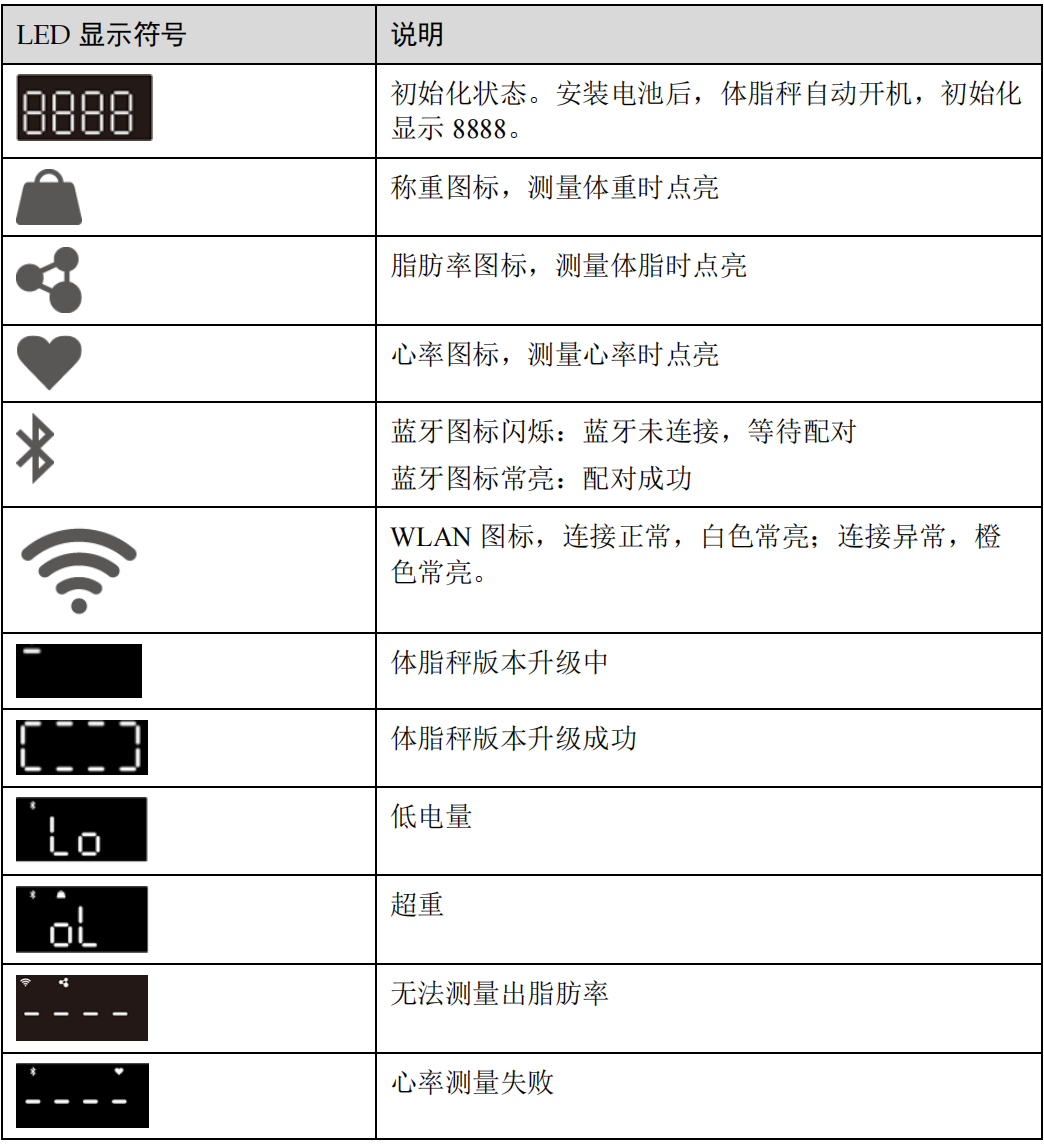


**4、如果没有称重成功，怎么处理？**

**
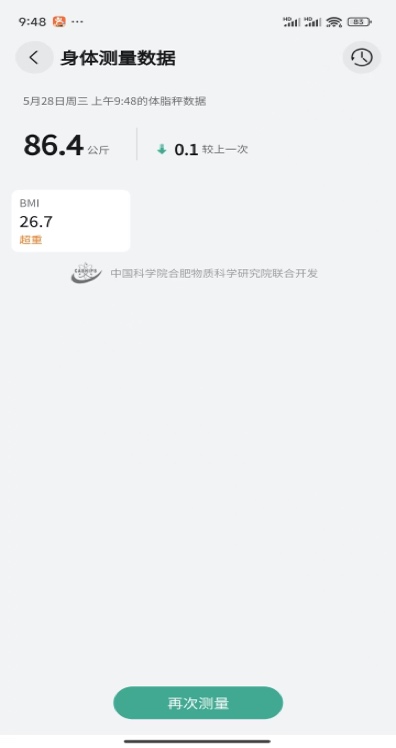
**

**
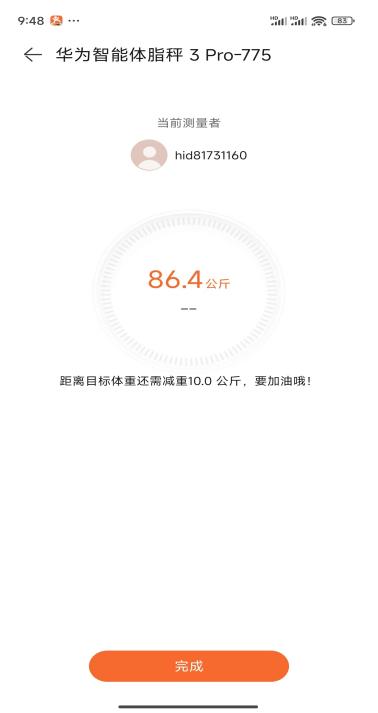
**

**如果只显示了体重和体脂率，可能提示没有摆好姿势，体重秤导电有问题。**

1. 排查是否有闪红色👣图标，有则代表导电问题。
2. 体重秤和手机可能未连接成功，需要开关蓝牙再尝试连接。
3. 可能不除外脚和手皮肤干燥问题，可在洗手洗脚后擦净，尝试重新测量。
4. 若尝试上述操作后仍无法成功，则可以拆卸体重秤电池重新安装。
