# Supplementary material for: Effectiveness and Feasibility of Self-Monitoring for Weight Management in Individuals With Mental Disorders Using Digital Intervention: Protocol for a Stepped-Wedge Cluster Randomized Trial (“SWIM” Study)
Source: JMIR Res Protoc. 2026 Apr 27;15:e78420. doi: 10.2196/78420 (PMC13120533; doi:10.2196/78420)
Supplement: Multimedia Appendix 2 [file resprot-v15-e78420-s002.doc]

|  | **STUDY PERIOD** | | | | | |  |  |
| --- | --- | --- | --- | --- | --- | --- | --- | --- |
|  | **Enrolment** | **Allocation** | **Post-allocation** | | | |  |  |
| **TIMEPOINT**** | **-t_1_** | **0m** | **1m** | **2m** | **3m** | **4m** | **5m** | **6m** |
|  |  | **V1** | **V2** | **V3** | **V4** |  |  | **V5** |
| **v5ENROLMENT:** | | | | | | | | |
| **Eligibility screen** | X |  |  |  |  |  |  |  |
| **Informed consent** | X |  |  |  |  |  |  |  |
| **Baseline**  **Assessments**  **(measures listed below assessments)** | X |  |  |  |  |  |  |  |
| **Allocation** |  | X |  |  |  |  |  |  |
| **INTERVENTIONS: Enhanced monitoring and lifestyle guidance** | | | | | | | | |
| **Cohort 1** |  |  | X | X |  |  |  |  |
| **Cohort 2** |  |  | X | X | X | X |  |  |
| **ASSESSMENTS:** | | | | | | | | |
| **Clinical**  **demographics** | X |  |  |  |  |  |  |  |
| **Height** | X |  |  |  |  |  |  |  |
| **Weight**  **self-monitoring** | X | X | Every week | | | | | |
| **Dietary dairy** | X | X | Every day | | | | | |
| **Psychiatric medication** | X |  | X | X | X |  |  | X |
| **Physical examination** | X |  | X | X | X |  |  | X |
| **Laboratory Tests** |  |  | X | X | X |  |  | X |
| **ECG, Hcg test** | X |  |  |  |  |  |  | X |
| **Self-reported scales** |  | | | | | | | |
| VAS | X |  | X | X | X |  |  | X |
| SDS | X |  | X | X | X |  |  | X |
| Q-LES-Q-SF | X |  | X | X | X |  |  | X |
| CFS-11 | X |  | X | X | X |  |  | X |
| PSS | X |  | X | X | X |  |  | X |
| ASEX | X |  | X | X | X |  |  | X |
| BPRS-4 | X |  | X | X | X |  |  | X |
| PHQ-9 | X |  | X | X | X |  |  | X |
| GAD-7 | X |  | X | X | X |  |  | X |
| MDQ | X |  | X | X | X |  |  | X |
| **Adverse events** |  |  | X | X | X | X | X | X |
| **Concomitant medication** |  |  | X | X | X | X | X | X |

** For participants who discontinue the study early, a final visit should be conducted with assessments equivalent to those scheduled for the end of Week 24.*

*** Physical examinations should include measurement of pulse, seated blood pressure, body weight, and documentation of abnormal findings in the head and face, skin, lymph nodes, eyes, ears, nose, throat, oral cavity, respiratory system, abdomen, cardiovascular system, musculoskeletal system, and nervous system.*

**** Laboratory tests include a 26-item biochemical panel, fasting insulin, C-reactive protein (CRP), five-item sex hormone panel, five-item thyroid function panel, and blood levels of mood stabilizers and antipsychotics (measured at baseline, at Visit 5, and whenever a medication dose is adjusted). Urine pregnancy testing should be performed for women of childbearing potential.*

*****Abbreviations of self-reported scales: VAS – Visual Analog Scale for weight loss motivation; SDS – Sheehan Disability Scale; Q-LES-Q-SF – Quality of Life Enjoyment and Satisfaction Questionnaire-Short Form; CFS-11 – Chalder Fatigue Scale; PSS – Perceived Stress Scale; ASEX – Arizona Sexual Experiences Scale; BPRS-4 – Brief Psychiatric Rating Scale, 4-item version; PHQ-9 – Patient Health Questionnaire-9; GAD-7 – Generalized Anxiety Disorder-7; MDQ – Mood Disorder Questionnaire.*
