# Supplementary material for: Effectiveness and Feasibility of Self-Monitoring for Weight Management in Individuals With Mental Disorders Using Digital Intervention: Protocol for a Stepped-Wedge Cluster Randomized Trial (“SWIM” Study)
Source: JMIR Res Protoc. 2026 Apr 27;15:e78420. doi: 10.2196/78420 (PMC13120533; doi:10.2196/78420)
Supplement: Multimedia Appendix 3 [file resprot-v15-e78420-s003.docx]

智能体重秤及辅助干预用户体验访谈量表

参与者基本信息

参与者ID：______

访谈日期：______

访谈者：______

第一部分：设备使用体验

1. 您觉得这款智能体重秤的外观设计如何？

- 非常不喜欢

- 不太喜欢

- 喜欢

- 非常喜欢

2. 您认为体重秤的尺寸大小是否合适？

- 太小了

- 有点小

- 正好合适

- 有点大

- 太大了

3. 使用这款体重秤时，您感到舒适吗？

- 非常不舒适

- 不太舒适

- 舒适

- 非常舒适

4. 您觉得体重秤的操作是否简单方便？

- 非常复杂

- 有点复杂

- 简单

- 非常简单

5. 您是否经常忘记使用体重秤进行定期称重？

- 总是忘记

- 有时忘记

- 很少忘记

- 从不忘记

6. 您是否愿意继续使用这款体重秤？

- 非常不愿意

- 不太愿意

- 愿意

- 非常愿意

7. 关于体重秤的使用体验，您还有什么想补充的吗？______

第二部分：APP使用体验

8. 您觉得配套APP的界面设计如何？

- 非常不友好

- 不太友好

- 友好

- 非常友好

9. 记录饮食和体重的功能是否容易操作？

- 非常困难

- 有点困难

- 容易

- 非常容易

10. 您是否经常使用APP记录饮食信息？

- 从不使用

- 很少使用

- 经常使用

- 每天使用

11. APP提供的饮食建议和健康数据分析对您有帮助吗？

- 完全没有帮助

- 帮助不大

- 有帮助

- 非常有帮助

12. 您是否愿意通过APP与医生或健康管理师分享您的健康数据？

- 非常不愿意

- 不太愿意

- 愿意

- 非常愿意

13. 关于APP的使用体验，您还有什么想补充的吗？______

第三部分：干预效果评估

14. 对您做了生活方式干预后，您对自身健康状况的了解是否有提高？

- 完全没有

- 有一点

- 有明显提高

- 有很大提高

15. 这种干预措施是否帮助您改善了健康行为（如规律称重、注意饮食等）？

- 完全没有

- 有一点

- 有明显帮助

- 有很大帮助

16. 您认为定期随访的间隔时间是否合适？

- 太频繁

- 有点频繁

- 正好合适

- 间隔太长

17. 随访内容对您的健康管理有帮助吗？

- 完全没有

- 有一点

- 有帮助

- 非常有帮助

18. 您会向亲友推荐这样的干预减重方式吗？

- 绝对不会

- 可能不会

- 可能会

- 一定会

19. 关于整体干预效果，您还有什么想分享的吗？______

第四部分：改进建议

20. 您觉得这套智能健康管理设备最需要改进的方面是什么？______

21. 您希望增加哪些功能或服务？______

22. 您对这套健康管理系统的其他建议：______

感谢您的参与和宝贵意见！
