# Supplementary material for: Effectiveness and Feasibility of Self-Monitoring for Weight Management in Individuals With Mental Disorders Using Digital Intervention: Protocol for a Stepped-Wedge Cluster Randomized Trial (“SWIM” Study)
Source: JMIR Res Protoc. 2026 Apr 27;15:e78420. doi: 10.2196/78420 (PMC13120533; doi:10.2196/78420)
Supplement: Multimedia Appendix 4 [file resprot-v15-e78420-s004.docx]

1 month (n= )

Lost to follow up (n= )

Withdrawn (n= )

2 months (n= )

Lost to follow up (n= )

Withdrawn (n= )

3 months (n= )

Lost to follow up (n= )

Withdrawn (n= )

6 months (n= )

Primary outcome data (n= )

Lost to follow up (n= )

Withdrawn (n= )

1 month (n= )

Lost to follow up (n= )

Withdrawn (n= )

2 months (n= )

Lost to follow up (n= )

Withdrawn (n= )

3 months (n= )

Primary outcome data (n= )

Lost to follow up (n= )

Withdrawn (n= )

6 months (n= )

Primary outcome data (n= )

Lost to follow up (n= )

Withdrawn (n= )

Follow up

APP and smart weight scale setup (n= )

Technical failure (n= )

Completed weighing at baseline (n= )

APP and smart weight scale setup (n= )

Technical failure (n= )

Completed weighing at baseline (n= )

Enrollment

Allocation

Cluster 1 (N=3)

Allocation to intervention (n= )

Cluster allocation (median=, range= )

Cluster 2 (N=3)

Allocation to intervention (n= )

Cluster allocation (median=, range= )

Consented (n= )

Approached for consent (n= )
