# Supplementary material for: Effectiveness and Feasibility of Self-Monitoring for Weight Management in Individuals With Mental Disorders Using Digital Intervention: Protocol for a Stepped-Wedge Cluster Randomized Trial (“SWIM” Study)
Source: JMIR Res Protoc. 2026 Apr 27;15:e78420. doi: 10.2196/78420 (PMC13120533; doi:10.2196/78420)
Supplement: Checklist 1 [file resprot-v15-e78420-s005.docx]

**The TIDier Checklist**

**1. Brief Name**

"Smart Devices Based Multicomponent Lifestyle Enhancement Intervention for Weight Management" Page 1

**2. Why**

To promote sustainable weight loss through structured self-monitoring, evidence-based lifestyle guidance, and behavioral reinforcement, tailored for SMI patients with varying intervention timelines. Page 3

**3. What (Materials)**

• Devices: Huawei Pro 3 scale (provided to participants).

• Digital Tools: Huawei Health App (configured for body composition tracking, dietary logging, and goal setting).

• Educational Content: Standardized dietary/nutrition tips, exercise guidelines, and motivational messages delivered via WeChat/SMS. Page 7, page 14

**4. Procedure and intervention**

App and scale provided during a 2-week pre-intervention transition period; Generic dietary/exercise materials accessible in-app; personalized plans available upon request.

• *Self-Monitoring:* Weekly weigh-ins (data auto-uploaded to app; participants submit screenshot confirmation).

• *Dietary Intervention:*

a. Calorie-deficit goals set via app (0.5–1 kg/week weight loss target).

b. Encouraged daily food logging; non-personalized dietary tips sent biweekly (WeChat/SMS).

c. Option for personalized nutritionist consultations.

• *Exercise Intervention:*

a. Generic weekly activity suggestions (e.g., 150–300 min aerobic exercise + 2–3 resistance sessions).

b. Option for personalized plans.

• *Behavioral Reinforcement:*

a. Automated reminders (WeChat/SMS): Weekly weigh-in prompts, biweekly diet/exercise tips.

b. Encouragement messages (e.g., adherence praise, progress statistics).

c. Escalation protocol for non-adherence (reminders → counselor outreach → in-person support).

Page 6-8

**5. Who Provided**

• Intervention Providers: Research staff (trained in app setup), certificate nutritionists and sports physician (for personalized plans), and automated systems (WeChat/SMS delivery).

• Training: Participants taught app/module usage during the 2-week transition.

Page 7-8

**6. How (Delivery)**

• Modes: Hybrid (digital: app/WeChat/SMS; optional in-person for escalated support).

• Format: Individualized, with generic content and optional personalized components.

Page 8

**7. Where**

• Location: Remote (self-monitoring and digital delivery) + clinical settings (for lab tests and scale filling)

**8. When and How Much**

• Timing:

a. Batch 1: Starts at Month 3 (4-month intervention).

b. Batch 2: Starts at Month 5 (2-month intervention).

• Frequency:

a. Weigh-ins: Weekly.

b. Dietary/exercise tips: Biweekly.

c. Reinforcement messages: ≥3/week.

Page 5-8

**9. Tailoring**

• Planned:

a. Goals adjusted to baseline BMI/preferences.

b. Optional personalization for diet/exercise.

• Actual: Generic content delivered uniformly; personalization upon request.

Page 7-8

**10. Modifications**

• Not applicable during the study.

**11. How well planned**

• Adherence Tracking: Monthly weigh-in counts.

• Strategies: Automated reminders, escalation protocol.

**12.How well actual**

• Not applicable during the study.
