# Supplementary material for: Effectiveness and Feasibility of Self-Monitoring for Weight Management in Individuals With Mental Disorders Using Digital Intervention: Protocol for a Stepped-Wedge Cluster Randomized Trial (“SWIM” Study)
Source: JMIR Res Protoc. 2026 Apr 27;15:e78420. doi: 10.2196/78420 (PMC13120533; doi:10.2196/78420)
Supplement: Checklist 2 [file resprot-v15-e78420-s006.doc]

|  | **STUDY PERIOD** | | | | | |  |  |  |  |
| --- | --- | --- | --- | --- | --- | --- | --- | --- | --- | --- |
|  | **Enrolment** | **Allocation** | **Post-allocation** | | | |  |  |  |  |
| **TIMEPOINT**** | **-t_1_** | **0m** | **1m** | **2m** | **3m** | **4m** | **5m** | **6m** | **7m** | **8m** |
| **ENROLMENT:** | | | | | | | | |  |  |
| **Eligibility screen** | X |  |  |  |  |  |  |  |  |  |
| **Informed consent** | X |  |  |  |  |  |  |  |  |  |
| **Baseline**  **Assessments**  **(measures listed below assessments)** | X |  |  |  |  |  |  |  |  |  |
| **Allocation** |  | X |  |  |  |  |  |  |  |  |
| **INTERVENTIONS: Enhanced monitoring and lifestyle guidance** | | | | | | | | |  |  |
| **Cohort 1** |  |  | X | X |  |  |  |  |  |  |
| **Cohort 2** |  |  | X | X | X | X |  |  |  |  |
| **ASSESSMENTS:** | | | | | | | | |  |  |
| **Clinical**  **demographics** | X |  |  |  |  |  |  |  |  |  |
| **Height** | X |  |  |  |  |  |  |  |  | X |
| **Weight**  **self-monitoring** | X |  | X |  | X |  | X |  |  | X |
| **Dietary dairy** | X |  | X |  | X |  | X |  |  | X |
| **Psychiatric medication** | X |  | X |  | X |  | X |  |  | X |
| **Physical examination** | X |  | X |  | X |  | X |  |  | X |
| **Laboratory Tests** |  |  | X |  | X |  | X |  |  | X |
| **ECG, Hcg test** | X |  |  |  |  |  |  |  |  | X |
| **VAS** | X |  | X |  | X |  | X |  |  | X |
| **SDS** | X |  | X |  | X |  | X |  |  | X |
| **Q-LES-Q-SF** | X |  | X |  | X |  | X |  |  | X |
| **CFS-11** | X |  | X |  | X |  | X |  |  | X |
| **PSS** | X |  | X |  | X |  | X |  |  | X |
| **ASEX** | X |  | X |  | X |  | X |  |  | X |
| **BPRS-4** | X |  | X |  | X |  | X |  |  | X |
| **PHQ-9** | X |  | X |  | X |  | X |  |  | X |
| **GAD-7** | X |  | X |  | X |  | X |  |  | X |
| **MDQ** | X |  | X |  | X |  | X |  |  | X |
| **Adverse events** |  |  | X |  | X |  | X |  |  | X |
| **Concomitant medication** |  |  | X |  | X |  | X |  |  | X |

*Recommended content can be displayed using various schematic formats. See SPIRIT 2013 Explanation and Elaboration for examples from protocols.

**List specific timepoints in this row.
