# Supplementary material for: Effectiveness and Feasibility of Self-Monitoring for Weight Management in Individuals With Mental Disorders Using Digital Intervention: Protocol for a Stepped-Wedge Cluster Randomized Trial (“SWIM” Study)
Source: JMIR Res Protoc. 2026 Apr 27;15:e78420. doi: 10.2196/78420 (PMC13120533; doi:10.2196/78420)
Supplement: Checklist 3 [file resprot-v15-e78420-s007.docx]

CONSORT 2010 Extension for Stepped-Wedge Cluster Randomised Trials (SW-CRT) Checklist

| Item No | Section/Topic | Item Description | Reported in Section/Page [Exact Text from Manuscript] |
| --- | --- | --- | --- |
| Title and Abstract |  |  |  |
| 1a | Title | Identification as a stepped-wedge cluster randomised trial in the title. | Title Page: "...A Stepped-Wedge Cluster Randomized Trial ('SWIM' Study)" |
| 1b | Abstract | Structured summary of trial design, methods, results, and conclusions. | Abstract,Page 1: "Methods: This single-center, open-cohort stepped-wedge cluster randomized trial (SW-CRT) with a batched rollout will recruit 204 patients from six psychiatric units." |
| Introduction |  |  |  |
| 2a | Background | Scientific background and explanation of rationale. | Page 2-3: "Patients with severe mental illness (SMI) face a 2-3-fold higher prevalence of obesity... primarily the metabolic side effects of antipsychotic medications... Digital health technologies have emerged as a particularly promising avenue..." |
| 2b | Objectives | Specific objectives or hypotheses. | Page 5: "The primary objective is to evaluate the effectiveness of the digital intervention on body weight reduction... Secondary objectives focus on capturing granular metabolic improvements and assessing implementation feasibility." |
| Methods |  |  |  |
| 3a | Trial design | Description of trial design including allocation ratio. | Study Design, Page 6-7: "This study utilizes a stepped-wedge cluster randomized trial (SW-CRT) with a batched rollout across six psychiatric units... randomized into two waves (3 units per wave)..." |
| 3a-1 | SW-CRT design | Identification of the trial as a stepped-wedge design, including sequences and steps. | Study Design, Page 6-7: "This study utilizes an open-cohort stepped-wedge design with a 2-month step duration... Crossover is implemented in two waves: Batch 1 (Wave 1)... Batch 2 (Wave 2)..." |
| 3a-2 | SW-CRT design | Definition of the cluster. | Study Design, Page 6-7: "...defining each cluster as a care unit 'Clinical Management Team' rather than a fixed physical location." |
| 3a-3 | SW-CRT design | Whether the trial is cross-sectional, closed-cohort, or open-cohort. | Study Design, Page 6-7: "This study utilizes an open-cohort stepped-wedge design..." |
| 4a | Eligibility | Eligibility criteria for participants. | Patient recruitment, Page 8: "Patients are considered for referral if they meet three prerequisites: (1) a BMI ≥24 kg/m²; (2) a demonstrated interest in weight management; and (3) a confirmed stable clinical phase..." |
| 4a-1 | Eligibility | Eligibility criteria for clusters. | Study Design, Page 6: "...six psychiatric units at a tertiary hospital in Beijing." |
| 5 | Interventions | The interventions for each group with sufficient details to allow replication. | Intervention Program,Page 10-11: "Participants are provided with a Huawei Pro 3 Bluetooth-enabled smart scale and the Huawei Health App (v10.0.2)... Core activities include weekly weigh-ins, daily dietary logging, and biweekly educational modules..." |
| 6a | Outcomes | Pre-specified primary and secondary outcome measures. | Outcome, Page 11: "Primary Outcome: The primary effectiveness outcome is the proportion of participants achieving ≥5% weight reduction from baseline, assessed at the end of the 8-month study period (Month 8)." |
| 7a | Sample size | How sample size was determined. | Sample Size, Page 14: "...a total sample of 204 participants (34 per unit) provides 82% power (α=0.05, two-tailed) assuming a conservative intra-cluster correlation coefficient (ICC) of 0.10." |
| Randomisation |  |  |  |
| 8a | Sequence generation | Method used to generate the random allocation sequence. | Study Design, Page 7: "The six units were randomized into two waves (3 units per wave) using a matched-pair constrained randomization approach." |
| 9 | Allocation concealment | Mechanism used to implement the random allocation sequence. | Study Design, Page 7: "Allocation was managed by an independent biostatistician via REDCap and remained concealed from the recruitment staff and participants." |
| 10 | Implementation | Who generated the sequence, who enrolled, and who assigned. | Study Design & Intervention Program (Page 7- 10): "Allocation was managed by an independent biostatistician... Treating psychiatrists identify potential participants... crossover dates were disclosed to each ward’s implementation team only two weeks prior..." |
| 11a | Blinding | If done, who was blinded and how. | Study Design, Page 7: "...remained concealed from the recruitment staff and participants. Crossover dates were only disclosed... two weeks prior... to minimize anticipation bias." |
| Statistical Methods |  |  |  |
| 12a | Statistical methods | Statistical methods used to compare groups. | Statistical Plans, Page 14-16: "For the primary binary outcome (≥5% weight loss), a Generalized Estimating Equations (GEE) model... For continuous secondary outcomes... we will employ a Linear Mixed-Effects Model." |
| 12a-1 | Statistical methods | How clustering and time effects were taken into account. | Covariate Adjustment and Confounder Control, Page 15-16: "To account for the nested cluster structure and secular time trends... intervention exposure duration (time since crossover) will be included as a time-varying covariate..." |
| Other Information |  |  |  |
| 23 | Registration | Registration number and name of trial registry. | Abstract, Page 2: "Trial registration: ClinicalTrials.gov NCT05866107. Registered on Aug 29, 2023." |
| 25 | Funding | Sources of funding and other support. | Funds, Page 19: "The study was funded by..." |
